# Rapid Metagenomic Diagnostics for Suspected Outbreak of Severe Pneumonia

**DOI:** 10.3201/eid2006.131526

**Published:** 2014-06

**Authors:** Nicole Fischer, Holger Rohde, Daniela Indenbirken, Thomas Günther, Kerstin Reumann, Marc Lütgehetmann, Thomas Meyer, Stefan Kluge, Martin Aepfelbacher, Malik Alawi, Adam Grundhoff

**Affiliations:** University Medical Centre Hamburg–Eppendorf, Hamburg, Germany (N. Fischer, H. Rohde, M. Lütgehetmann, T. Meyer, S. Kluge, M. Aepfelbacher, M. Alawi);; German Center for Infection Research, Hamburg (N. Fischer, A. Grundhoff);; Heinrich Pette Institute Leibniz Institute for Experimental Virology, Hamburg (D. Indenbirken, T. Günther, K. Reumann, M. Alawi, A. Grundhoff)

**Keywords:** atypical severe pneumonia, acute respiratory distress syndrome, ARDS, Chlamydophila psittaci, bacteria, respiratory infections, rapid metagenomic diagnostics, infectious disease outbreak, high-throughput nucleotide sequencing, northern Germany

**To the Editor:** Recent outbreaks of severe pneumonia or acute respiratory distress syndrome (ARDS) have attracted much public interest. Given current awareness levels, clinical personnel and health officials must rapidly and adequately respond to suspected outbreaks to prevent public disturbances. We report a case that highlights the potential of next-generation sequencing (NGS) to complement conventional diagnostics in such scenarios.

On March 29, 2013, a police officer (patient 1) was admitted to the emergency department of the University Medical Centre Hamburg–Eppendorf in Hamburg, Germany, because of ARDS. The patient was given mechanical ventilation; all diagnostic test results for pathogens commonly known to cause pneumonia were negative (www.virus-genomics.org/supplementaries/EID1406.pdf). Although treatment with antimicrobial drugs was immediately initiated, the patient died 6 days later of multiple organ failure.

On April 5, a second police officer (patient 2) from the same county was admitted to the same emergency department because of ARDS. He was transferred to the intensive care unit and given mechanical ventilation. Similar to the situation for patient 1, diagnostic test results were negative, prompting the news media to suspect an outbreak of a novel or mutated virus (*1**,**2*). Especially because of simultaneous outbreaks of avian influenza and infections with Middle East respiratory syndrome coronavirus in other parts of the world, these reports caused serious concern among the public and health officials.

After the death of patient 1 and hospitalization of patient 2, we subjected nucleic acids extracted from bronchoalveolar lavage (BAL) specimens from both patients to NGS by using a MiSeq sequencer (www.illumina.com/systems.ilmn). To enable rapid and unbiased detection of bacterial and viral agents, we did not enrich specific sequences. The entire workflow (www.virus-genomics.org/supplementaries/EID1406.pdf) was completed within 50 hours.

First-line analysis clearly identified *Chlamydophila psittaci* in the RNA sample from patient 2, but no sequences of obvious pathogenic origin were detected in samples from patient 1 (Figure). *C. psittaci*, an intracellular bacterium, can be transmitted by inhaling aerosolized secretions or feces from infected birds (*3*). Person-to-person transmission of this bacterium is rare (*4**,**5*). Ornithosis, a disease characterized by severe pneumonia and influenza-like symptoms, might develop in persons infected with this bacterium. Because of the rarity of the disease, standard diagnostic panels usually do not include *C. psittaci*. After 11 days of antimicrobial drug treatment, the condition of patient 2 improved, and the patient was transferred to a general hospital ward.

**Figure:**
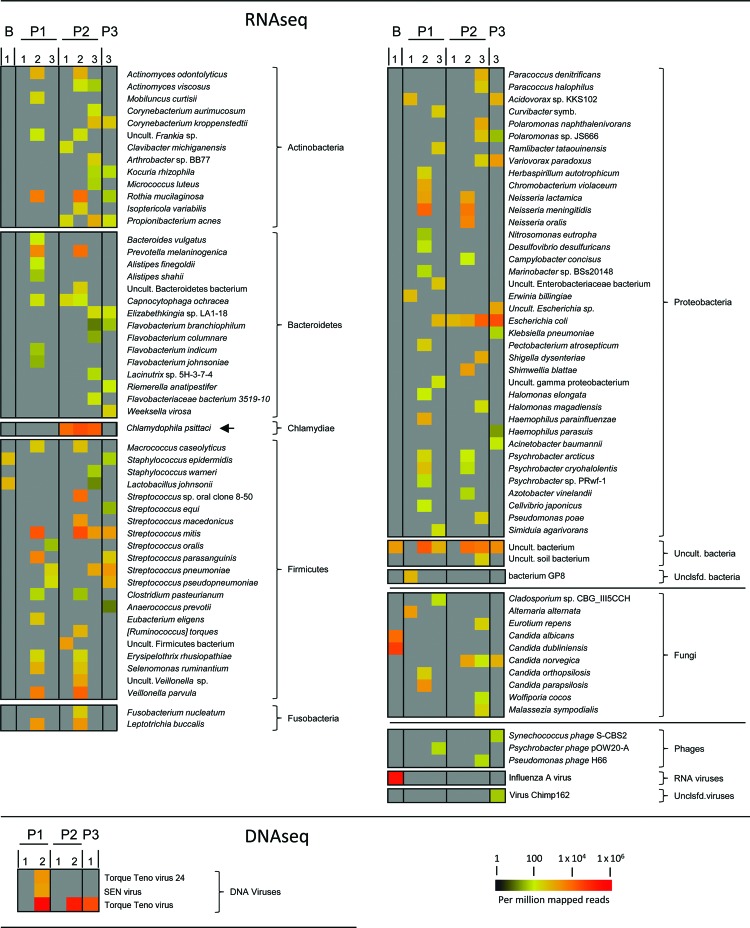
Next-generation sequencing of RNA (RNaseq) and DNA (DNaseq) isolated from bronchoalveolar lavage (BAL) samples from 3 patients with severe pneumonia, northern Germany. Shown are data from BLASTN (http://blast.ncbi.nlm.nih.gov/Blast.cgi) analysis of de novo assembled sequence contigs (www.virus-genomics.org/supplementals/EID1406.pdf). Relative abundance of contig reads mapping to bacterial, fungal, or viral species is indicated by a heat map (scale bar). Gray indicates that no reads were detected. Diagnostic samples were obtained from 3 patients (lanes P1, P2, and P3). Lane B, control BAL sample (analyzed by using RNaseq only) from an influenza patient; lane1, MS: analysis on the Illumina MiSeq platform (www.illumina.com/systems.ilmn); lane 2, HS: analysis on the Illumina HiSeq platform (www.illumina.com/systems.ilmn); lane 3, HS dpl., RNA samples depleted of human rRNA before analysis on a HiSeq instrument. *Chlamydophila psittaci*, which was unequivocally detected in all samples from patient 2 but not in samples from the other patients, is indicated by an arrow. Symb., symbiont; Uncult., uncultured; Unclsfd., unclassified; SEN virus, strain of Torque teno virus.

On April 29, a third police officer (patient 3) who had been sharing office space with patient 2, came to the hospital with symptoms of pneumonia. After antimicrobial drug treatment, his condition rapidly improved and the patient was discharged on day 7.

At admission of patient 3, samples from all 3 patients were analyzed at greater read depth by using a HiSeq system. RNA sequencing identified commensal bacteria in all samples, but *C. psittaci* was present only in samples from patient 2 (Figure). Likewise, subsequent high-throughput 16S rRNA sequencing (*6*), PCR, and serologic analysis unequivocally identified a *C. psittaci* infection in patient 2, but not in patients 1 or 3 (www.virus-genomics.org/supplementaries/EID1406.pdf).

We did not detect viral pathogens in any samples. At the DNA level, most nonhost reads originated from nonpathogenic single-stranded DNA anelloviruses (*7*). No RNA viruses were found, although influenza A(H3N2) virus was readily identified in a MiSeq analysis of a control BAL sample from a patient with a diagnosis of influenza A (PCR cycle threshold 32) (Figure). Furthermore, pairwise BLAST analysis (http://blast.ncbi.nlm.nih.gov/Blast.cgi) did not reveal the presence of unknown sequence contigs that were shared among the patients, as would be expected in case of infection with a novel viral agent. Together with the confirmed *C. psittaci* infection in patient 2, the absence of a common pathogen signature strongly suggests that the cases were unrelated.

We used a comprehensive metagenomic approach to resolve cases suspected of representing an ongoing outbreak. The method used enabled diagnosis of a *C. psittaci* infection within a reasonable timeframe to allow for timely clinical intervention. These findings strongly suggest that NGS methods can complement conventional diagnostics (*8**–**10*) and also highlight their potential to aid clinical personnel and health agencies in making appropriate decisions during suspected outbreaks. Clearly, however, NGS-based methods will have to be further standardized and validated before their full potential in diagnostic settings can be realized.

The absence of pathogenic sequences in patients 1 and 3 might suggest that their clinical symptoms had noninfectious causes. However, although samples were collected during the acute phase of clinical symptoms, and despite our ability to detect an influenza A infection in controls, we cannot fully exclude the possibility that a potentially causative pathogen present at low levels might have evaded detection. Thus, systematic and correlative studies evaluating the sensitivity of NGS-based detection methods in different diagnostic entities are urgently needed.
